# Cyberbullying Prevention for Adolescents: Iterative Qualitative Methods for Mobile Intervention Design

**DOI:** 10.2196/25900

**Published:** 2021-08-27

**Authors:** Megan L Ranney, Sarah K Pittman, Isabelle Moseley, Kristen E Morgan, Alison Riese, Michele Ybarra, Rebecca Cunningham, Rochelle Rosen

**Affiliations:** 1 Center for Digital Health Brown University Providence, RI United States; 2 Rhode Island Hospital Providence, RI United States; 3 Center for Innovative Public Health Research San Clemente, CA United States; 4 University of Michigan Ann Arbor, MI United States; 5 Center for Behavioral and Preventive Medicine The Miriam Hospital Providence, RI United States

**Keywords:** adolescent, mobile health, digital health, cyberbullying, user-centered design, qualitative, mobile phone

## Abstract

**Background:**

Cybervictimization among adolescents is associated with multiple negative mental health consequences. Although pediatricians often screen for cyberbullying, validated and acceptable programs to reduce the frequency and impact of adolescent cybervictimization are lacking.

**Objective:**

This study uses *agile* qualitative methods to refine and evaluate the acceptability of a mixed-modality intervention, initiated within the context of usual pediatric care, for adolescents with a history of cyberharassment and cyberbullying victimization.

**Methods:**

Three groups of adolescents were successively recruited from an urban primary care clinic to participate in three consecutive iterations (1, 2, and 3) of the program, which consisted of a brief in-clinic intervention followed by 8 weeks of daily, automated SMS text messaging. After 2 weeks of messaging, iteration 1 (I1) participants completed semistructured interviews regarding intervention experiences. Participant feedback was evaluated via framework matrix analysis to guide changes to the program for iteration 2 (I2). Feedback from 2-week interviews of I2 participants was similarly used to improve the program before initiating iteration 3 (I3). Participants in all 3 iterations completed the interviews after completing the program (8 weeks). Daily response rates assessed participant engagement, and satisfaction questionnaires assessed acceptability.

**Results:**

A total of 19 adolescents (aged 13-17 years) reporting past-year cybervictimization were enrolled: 7 in I1, 4 in I2, and 8 in I3. Demographic variables included the following: a mean age of 15 (SD 1.5) years; 58% (11/19) female, 42% (8/19) male, 63% (12/19) Hispanic, 37% (7/19) non-Hispanic, 79% (15/19) people of color, and 21% (4/19) White. A total of 73% (14/19) self-identified as having a low socioeconomic status, and 37% (7/19) self-identified as lesbian, gay, or bisexual. The average past 12-month cybervictimization score at baseline was 8.2 (SD 6.58; range 2-26). Participant feedback was used to iteratively refine intervention content and design. For example, participants in I1 recommended that the scope of the intervention be expanded to include web-based *conflicts* and *drama*, rather than narrowly focusing on cyberbullying prevention. On the basis of this feedback, the I2 content was shifted toward more general de-escalation skills and bystander empowerment. Overall, 88.34% (940/1064) of the daily queries sent to participants across all 3 iterations received a reply. Participant satisfaction improved considerably with each iteration; 0% (0/7) of I1 participants rated the overall quality of Intervention to Prevent Adolescent Cybervictimization with Text message as *excellent*, compared to 50% (2/4) of I2 participants and 86% (6/7) of I3 participants. Engagement also improved between the first and third iterations, with participants replying to 59.9% (235/392) of messages in I1, compared to 79.9% (358/488) of messages in I3.

**Conclusions:**

This study shows the value of structured participant feedback gathered in an agile intervention refinement methodology for the development of a technology-based intervention targeting adolescents.

## Introduction

### Background

Cyberbullying, defined as the intentional harm of others through computers, cell phones, and other electronic devices, has become increasingly common [1]. Although rates of cyberbullying vary widely, from 6% to 72% depending on the study, approximately 25% of American adolescents have reported being victims of cyberbullying and web aggression (hereafter referred to as *cybervictimization*) in the past year [2,3]. Cybervictimization is strongly associated with negative consequences, including depressive symptoms and suicidality, posttraumatic stress, alcohol and other drug use, physical violence, and dating violence [4,5]. Although school-based cyberbullying prevention programs may be effective, many youths find it difficult to engage or retain in school-based prevention programs [6].

Almost 80% of adolescents have yearly well-child visits with their pediatrician [7]. Pediatricians play a long-recognized role in behavioral counseling [8]. The American Academy of Pediatrics recommends advising families about cybervictimization [9], but pediatricians lack both time and validated interventions [10]. An easy-to-use, clinically initiated program that enhances users’ protective skill sets (eg, emotion regulation and positive social support) against cybervictimization may be helpful [11] to pediatricians. Delivering preventive interventions through technology places fewer demands on staff time than in-person delivery, has inherently higher fidelity, and maybe lower cost [12]. KiVa, a school-based antibullying program that includes web-based components such as games, video clips, and infographics, has been shown to significantly reduce the frequency of cyberbullying and cybervictimization among Finnish youth [13]. Given the success of this technology-based approach, SMS text messaging is a logical medium for delivering interventions aimed at reducing cyberbullying and cybervictimization. Furthermore, our prior work indicates that delivering interventions in the same modality as bullying (ie, electronically) may increase intervention efficacy [14]. SMS text messaging is almost universally used by adolescents from all socioeconomic and racial or ethnic backgrounds [15]. SMS text message–augmented interventions are feasible, acceptable, and may be effective in reducing in-person fights [16-18]. Grounded in previous evidence that electronic interventions are effective for cyberbullying prevention and cybervictimization support and that SMS text messaging is a reliable and efficient way to deliver behavioral change interventions, the following clinical trial applies the technology of SMS text messaging to cyberbullying.

### Objective

In the larger project that serves as the use case for this manuscript, our aims are to develop, iteratively refine, and then pilot an SMS text message–based intervention to help adolescents recognize, cope with, and prevent cyberbullying. Our initial intervention prototype was developed from prior SMS text message–based interventions for physical violence and bullying, using existing cyberbullying prevention resources and expert consultation. To refine our prototype, we conducted hour-long qualitative interviews with 23 adolescents with past-year histories of cybervictimization and web-based conflict. Participants shared their own coping strategies for dealing with cyberbullying and offered constructive criticism of intervention content and design. Data from participant feedback were analyzed and used to update the structure and content of the intervention. In this study, we used qualitative methods—which classically occur as a discrete step in the research process—in 3 agile iterations to seek feedback from the intervention audience as the program is further refined. *Agile* methods are a development strategy used in software design, project management, manufacturing, and recently, health behavior research [19,20]. In agile methods, aspects of development occur collaboratively, instead of in isolation (as in traditional development), to incrementally build the product [20]. Iteration, a key component of the agile development process, facilitates usability testing. Review, planning, and testing can all occur iteratively. This paper describes methods used to gather, analyze, and use qualitative and quantitative data in real time for iterative intervention refinement of a cyberbullying intervention.

## Methods

### Study Setting and Recruitment

This study was conducted from July to November 2017 in a pediatric primary care clinic within an urban teaching hospital in the northeastern United States. Adolescents aged 13-17 years who presented with primary care (well-child and sick visits) were potentially eligible. Adolescents were excluded if they or their parents did not speak English, were in the custody of police or child protective services, had a diagnosis of intellectual or developmental disability, or did not have a parent or legal guardian present. After adolescent verbal assent and parent verbal permission, adolescents completed a screening survey on a tablet computer. Adolescents were eligible for participation if they screened positive for past-year cybervictimization [21], owned a cell phone with text messaging capabilities, and provided written assent and parental permission, per our local institutional review board’s request.

Three consecutive iterations of the SMS text messaging program were pilot-tested by adolescents; in each phase, recruitment continued sequentially until qualitative saturation was reached. First, 8 participants were enrolled for iteration 1 (I1) of the program. Enrolled participants completed a brief in-clinic intervention followed by up to 8 weeks of automated text messaging. After 2 weeks of receiving messages, I1 participants completed semistructured interviews that sought feedback on the program. Participant feedback was then analyzed via a framework matrix and used to guide changes to the content and structure of both the in-clinic intervention and the SMS text-messaging program. A second group consisting of 4 participants was recruited for iteration 2 (I2) to test the impact of these changes. Feedback from 2-week interviews of I2 participants was similarly used to improve the program before initiating iteration 3 (I3), for which a group of 9 participants was recruited. Participants in all 3 iterations were asked to complete an in-person semistructured interview after the receipt of text messages was concluded (8 weeks). This strategy shortened design-to-delivery time as per *generative* and *prototyping* phase of agile intervention development [20]. Participants in all iterations received a US $25 gift card at baseline, US $20 for completing the 8-week interview, US $40 for completing the follow-up survey, and US $10/month for the cost of text messaging or data use. The study materials and procedures were approved by the institutional review board.

### Intervention Structure and Content

Intervention to Prevent Adolescent Cybervictimization with Text message (iPACT) has two components: a brief (15-minute) in-clinic PowerPoint-guided session conducted by a trained, bachelors-level research assistant based on motivational interviewing and basic principles of cognitive behavioral therapy, a type of psychotherapy focused on strategies to identify and overcome destructive thought patterns (eg, using thoughts and actions to change feelings) and an 8-week, daily, automated, two-way text messaging curriculum. In addition to daily automated messages, participants could *pull* additional message content by texting keywords. Content was based on in-person and SMS text message–based violence prevention and cyberbullying prevention interventions [6,13,16-18]. Additional details, including the original structure of the intervention before the agile development process, are described elsewhere [14].

### Measures

#### Cyberbullying

The Cyberbullying Scale [21], used to determine study eligibility, is a validated 16-item self-report measure of past-year cybervictimization. Total scores are calculated by summing the participant responses to questions 3-16. Cronbach α was .88. Adolescents were eligible if they reported a frequency of *2=Sometimes* or more on any Cyberbullying Scale item.

#### Semistructured Interview

For I1 and I2 participants, semistructured qualitative interviews were completed after 2 weeks and 8 weeks of interaction with the intervention, respectively. Participants in I3 underwent 8-week interviews only. Interviews were conducted by a research assistant trained in qualitative data collection and interview facilitation. The interview agenda was designed to generate actionable data for intervention refinement by focusing on how participants perceived and responded to the intervention content. Discussions explored participants’ perceptions about, and usability of, key intervention components (in-person and SMS text message content; message tone, frequency, and applicability; daily queries; links to external content; and extra support messages). In the interviews, participants were asked to reflect on message wording and preferences; how, when, and why they engaged with the program; and what they were thinking about when they responded to daily messages. The interviews were digitally recorded. The interviewer completed a written debriefing after each interview.

#### Acceptability and Feasibility

Feasibility was determined by response rates to daily SMS text message queries, frequency of participant-requested extra support messages, and follow-up rates. To assess acceptability, the Client Satisfaction Questionnaire [22], a self-report measure (range 8-32) with high validity and internal consistency, was administered at the 8-week follow-up [22].

#### Demographics

Participants self-reported age, school grade, socioeconomic status (SES), race, ethnicity, birth sex, gender identity, and sexual orientation using validated measures [23-26].

### Analysis

A framework matrix analysis of qualitative interview data was used to continuously refine the intervention [27,28]. The framework matrix captured participant reactions to intervention components and their answers to the interview questions. Each participant’s comments were summarized in a matrix row; matrix columns represented the intervention components discussed in the interview. The interview facilitator listened to the audio recordings, summarizing participant comments in relevant cells. Verbatim quotes were included to provide context for the participants’ own words. To ensure credibility, a second analyst reviewed the matrix and audio for each interview, confirming the accuracy and completeness of the summaries. This data reduction technique quickly summarized qualitative information into a single usable source that was shared with coinvestigators and used in weekly study team meetings, allowing us to use the feedback from 2-week interviews of I1 participants to guide intervention refinement before I2 and the 2-week interviews of I2 participants before I3. At these team meetings, conflicting qualitative data were discussed, and a team consensus was reached on how it should be prioritized for intervention refinement. Notes indicating changes made based on the coinvestigator review of participant comments were added to the matrix to track changes.

Descriptive statistics were calculated for all quantitative measures. In accordance with prior work [29,30], acceptability was defined as 80% or greater scores on the Client Satisfaction Questionnaire, and feasibility was defined as 80% response to texts and 80% retention at the 8-week follow-up.

## Results

### Overview

Of the 121 adolescent patients who completed the study screening, 31 (25.6%) were eligible. Most enrollment refusals were because of *lack of time*, given the clinic setting. No otherwise eligible adolescents who completed the screening survey had to be excluded because of lack of parental permission.

Consistent with qualitative research sampling, we purposefully enrolled a diverse sample to capture potential outlier perspectives. A total of 8 participants enrolled in I1, 4 in I2, and 9 enrolled in I3. The mean age of all enrolled participants was 15 (range 13-17) years; the majority self-identified as people of color (15/19, 79%), Hispanic (12/19, 63%), female (11/19, 58%), and low SES (14/19, 73%). A total of 37% (7/19) of participants were identified as lesbian, gay, or bisexual. The average past 12-month cybervictimization score at baseline was 8.2 (SD 6.58; range 2-26).

Two participants withdrew (1 in I1 and 1 in I3) because they lost or broke their phones before starting the texts. In I1, all 7 participants completed the 2-week interview and 8-week survey; in I2, all 4 participants completed the 2-week interview and 3 completed the 8-week survey; in I3, 5 participants completed the 8-week interview and 7 of 8 completed the 8-week survey.

### Intervention Refinement: Qualitative Data

#### Overview

The key components of the intervention were (1) content, (2) tailoring, and (3) design and delivery. Qualitative data, as summarized in the framework matrix, were used to significantly revise each of these intervention components. The specifications of this process are described below; Table 1 provides additional details of these changes, including an example of the qualitative feedback given and the resulting changes made for each key topic.

**Table 1:** Exemplars of themes from framework matrix analysis, organized by topic and iteration designated by iteration 1, iteration 2, and iteration 3.

Topics addressed			Exemplar quotes		Examples of changes made
**General intervention content**					
	I1^a^: focus on general web-based drama and conflict rather than only on cyberbullying specifically	“...school drama and online drama kinda go hand in hand because if you were to have social media, you would of course have, like, most of your friends from school on social media.” [ID 29; 13 year; female]		The word cyberbullying was replaced with online drama on several slides of the PowerPoint used in the in-clinic session. The research assistant conducting the in-clinic intervention began framing the program as prevention for online drama and cyberbullying (rather than as a program about dealing with cyberbullying) and focused more on web-based drama in discussion The order of the messages was changed so that cyberbullying-specific content was emphasized during the last week of the texting program	
	I1: teens thought too much of the content was specific to cyberbullying and requested more general self-efficacy content	“Give something more like-like common sense. Cause nowadays people lack that so much.” [ID 2; 17 years; male]		A message offering possible responses to web-based bullies was replaced with a reminder that jokes or roasting can go too far and that teens should not be afraid to speak up in such situations Messages were added or changed to enhance self-efficacy; for example, “everyone is affected by drama” was changed to “you can make a difference”	
	I2^b^: participants liked random messages that were positive and inspirational	“...have, like, a nice inspirational quote like those, or, like, motivational quotes...I really liked them because it’s the..., it’s, like, the middle of the school day for me, so it’s something that gets me, like, to keep going.” [ID 12; 17 years; female]		Inspirational quotes from other teens such as “If it won’t matter in 5 years, don’t spend more than 5 minutes thinking about it” were used in the daily messages Additional random inspirational messages were added	
	I1, I2, I3^c^: participants thought positive content elevated their mood and also helped them avoid conflict	“Like, there was one day when I, like, woke up, and I was feeling very mad at this person on social media, and [the study text] was, like, something about...‘just think positive,’ and...something, ‘don’t go on there and be a bully.’ And I think it helped ‘cause I was gonna go on social media and talk to that person, but [the study text] it just made me think, oh, well that’s not gonna change anything. What she said is still what she said, so it kinda redirected me in a different path.” [ID 1; 15 years; female]		Messages such as “only have/keep good people on your page” were added to encourage participants to surround themselves with supportive friends	
	I3: teens suggested adding more content relating to stress and anxiety	“With anxiety, maybe you’re at, like, a big party, and you’re feeling really stressed out cause of all the people there.” [ID 4; 14 years; male]		Tips about changing thoughts and feelings were sent earlier in the program (previously scheduled for Weeks 6-7)	
**Tailoring**					
	I1: each participant defined cyberbullying differently and had unique experiences with it	“So cyberbullying is, like, when people, like, try to, like, get you online, right?” [ID 7; 15 years; female] “Some people be just, like, really, like, sittin’ at their computer hurtin’ people...It’s, like, sad, and, like, people, like...eventually they just gonna snap, and...be like, ‘Oh, why should I even be on, like, this earth anymore.’” [ID 31; 15 years; female] “I feel like once people get, like, really overwhelmed, they just kinda give up on everything, and that’s probably when they’ll jump to, like, social media...” [ID 12; 17 years; female]		The research assistant began tailoring the content to each participant based on the participant’s age and past-year versus past 2-month Cyberbullying Scale scores. The research assistant also incorporated each participants’ language into their intervention session	
	I2: participants wanted to provide their own strategies for making sense of intervention content rather than relying on confusing graphics in the in-clinic session	“Sometimes you can have a negative thought but then a smart action.” [ID 7; 15 years; female] “Like, stay positive...Like, don’t be a bully...I’m not a bully, so I was like, you know what, lemme just leave it alone.” [ID 17; 15 years; female]		Participants were asked to provide their own example for the research assistant’s explanation of the Thoughts-Feelings-Actions Triangle during the in-clinic session New intervention content was incorporated to allow participants to outline their own positive goals for social media	
	I2: participants were using different time frames over which to evaluate the daily cyberbullying SMS text message query because some received it in the morning and some in the afternoon	“Like, I know that there’s not gonna be drama at, like, 5:30 in the morning, so I kinda, like, play it back to the day, like, the night, before.” [ID 17; 15 years; female]		The language of the daily SMS text message drama question was changed from “Any drama today, yes or no?” to “Any drama in the past 24 hours, yes or no?” to make sure that every participant’s assessment period was the same length	
	I1 and I2: participants forgot about the on-demand messages and suggested sending a reminder	“I wish I knew or remembered that I could’ve texted back. I completely forgot. I didn’t know that I could’ve done that, and if I knew that I could’ve done that, I definitely would’ve texted back.” [ID 15; 14 years; female]		Additional reminders were added, for example, “Remember, if you ever want extra advice you can text us any time: ANGRY, SAD, HAPPY, or STRESSED.”	
	I1, I2, I3: participants generally felt the daily messages matched their day; however, they reported that on days with web-based drama, they wanted specific advice about their conflict	“If someone’s really having a bad day, they’ll get annoyed, but maybe just, like, one extra question, like, letting it all out in, like, one huge paragraph thing.” [ID 5; 13 years; female] “I feel like it’s good for you guys to, like, check up on us and, like, asking us, like, how our day is and how we feeling and thoughts and everything.” [ID 10; 14 years; male]		This suggestion was difficult to incorporate because the study was not designed for staff review of individual participants’ experiences; some examples of responses and actions in specific conflicts were added in website links in the texts	
**Design and delivery of the intervention**					
	I1: participants preferred that the messages sounded like they were coming from someone older than them, and they did not want them to sound too automated	“I wouldn’t trust it, kind of [if it were coming from someone my age]. ‘Cause it’s like – it’s kinda weird, ‘cause, like, they’re like me, and they also need help, but, for an adult, they’ve already been through a situation like this, so they know what to do on it.” [ID 1; 15 years; female] “I liked it because it was – it, like, it kinda felt like somebody, you know, cared to ask me how I was doing and how my day was.” [ID 16; 16 years; female]		A message that began with the rhetorical question, “How do you move on after online drama?” (which sounded like a computer) was rephrased to begin, “Some teens feel hopeless or sad after online drama.” The message “Useful tip (which you might know already): You can block or unfriend people who are bothering you online.” Was rephrased as “If someone is bothering you, you can block or unfollow them. We’ve got some great tips on controlling your social media page” to sound less curt and more sympathetic, like an adult or older sibling	
	I1, I2, and I3: participants would click on links only if they felt relevant, quick, and interesting	“[I clicked on a link] when I wasn’t having a good day, and I needed it, like, I felt like I needed advice or a video or anything to just, to distract myself from everything else and to help me.” [ID 15; 14 years; female] “I think they’re helpful, like, having the link there for you if you don’t – if you, like, need more help or something. Like, if I needed more tips, or I felt I needed more tips, I would click on the links, but in some days when, like, I didn’t need any more tips, like, that was great enough.” [ID 1; 15 years; female)		The advertising industry principle of clickbait was used in editing several of the messages to entice participants to click on the links more often	
	I2 and I3: participants requested app-based intervention and messaging instead of in-person interventions and texting	“It (an app) would be easier because...not a lot of teenagers will answer your text messages...everybody uses, like, Snapchat and everything more than that, so more people are likely to go on to the app than to go onto the text messages.” [ID 11; 14 years; female]		This suggestion was difficult to incorporate because of inherent study design issues	
	I2 and I3: participants recommended the program to friends	“I told one of my friends about it...cause, like, she’s always involved in drama. I told her, I was like, ‘hey, you should try this,’ and I showed her one of the websites. She liked it, and she asked me, like, how did I get it on my phone.” [ID 16; 16 years; female] “All my friends who actually, like, would need it or like it, and they were like, ‘oh that’s cool I wish I got that.’” [ID 15; 14 years; female] “It’s a cool program...a lot of people (my friends) were interested...I wish I could still do it.” [ID 12; 17 years; female]		The program’s final message was changed to end with, “Remember all you’ve learned these past 8 weeks and pass it on to your friends!”	

^a^I1: iteration 1.

^b^I2: iteration 2.

^c^I3: iteration 3.

#### General Intervention Content

In I1 qualitative interviews (Table 1), participants recommended discussing web-based *conflicts* and *drama* rather than narrowly focusing on cyberbullying prevention. On the basis of this feedback, I2 content was changed to discuss *web-based drama* in addition to *cyberbullying* and to provide more general de-escalation skills and bystander empowerment. In-person brief intervention language was also changed (eg, instead of asking “how many other teens do you think have been cyberbullied?” teens were asked to report their own daily experience with *web-based drama*). Cyberbullying-specific statistics were moved to the later weeks of the text program. Content was reframed to enhance the self-efficacy of those witnessing cyberbullying, as well as those experiencing it. These changes garnered positive feedback from users in I2 and I3.

In I2 qualitative interviews, participants further emphasized the importance of empowerment: teens said they wanted content that would help them take positive actions, boost confidence, and know how to intervene in web-based conflicts. They suggested content focused on using social media for good, sharing examples of supportive content they had found on the web. In response to this feedback, I3 in-clinic sessions incorporated a discussion of how healthy social media habits could help teens reach long-term personal goals, and positive rather than negative valence was emphasized. After these changes, feedback on content in I3 was overall positive; the majority of I3 participants described messages as *educational, motivational, helpful,* and *inspirational*. Numerous participants described how intervention messages motivated them to change their behavior by targeting their thoughts and feelings or to improve their emotional state by taking action.

#### Intervention Tailoring

In I1, the participants wanted greater personalization of both portions of the intervention. Accordingly, the in-person intervention was significantly reworked (Table 1). For example, the participants’ personal definitions of cyberbullying were incorporated. Specific changes were made to in-person session graphics to allow for greater incorporation of participant feedback. The SMS text message algorithms were also adjusted. For instance, a baseline intervention feature provided an option to text the program in the moment and to receive on-demand support for certain mood states; however, only 2 participants used this feature. In I1 and I2 qualitative interviews, most indicated that they simply forgot the feature existed and suggested sending a reminder. Consequently, multiple reminders of the on-demand feature were added to I3, and nearly all (n=7) participants used it.

Our initial design tailored messages based on each participant’s answers to 2 daily assessment questions. Participants were asked to rate their day (on a scale of 1=really bad to 5=great) and to indicate whether they experienced cyberbullying that day (yes or no). On the basis of feedback from I1 users, those questions were revised to ask about *drama or conflict on the web* rather than cyberbullying, and the qualifier *in the past 24 hours* was included because we found the recall period varied when *today* was the prompt. The language change was essential and effective: most interview participants (n=12) said the daily check-in made them feel as if *someone cared*. We were able to see increasing proportions of participants indicating that the daily message matched how they rated their day (3/7, 43% interview participants in I1; 3/4, 75% interview participants in I2; and 4/5, 80% interview participants in I3).

About half of all participants wanted the ability to give more information about their day, and several wanted to be able to get advice on specific instances of web-based drama. Human subject concerns about the collection of large amounts of free-text data from participants, ethical responsibilities to monitor and respond to them, budget, and staff limitations prevented the incorporation of this suggestion. Instead, links to websites and infographics were added after I2 to include specific conflict resolution examples. Although the addition was perceived as helpful, some I3 participants still wanted specific advice.

#### Design and Delivery of Intervention

**Figure 1:**
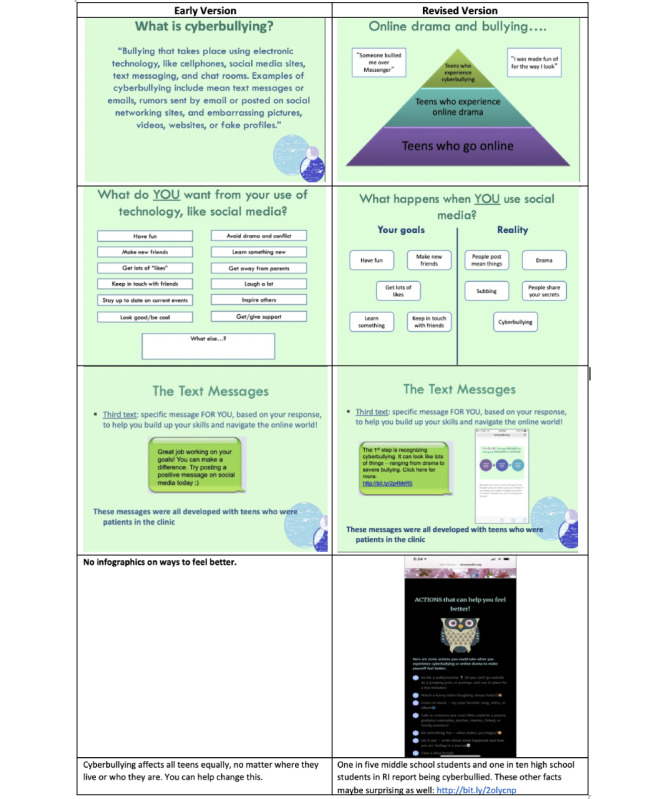
Exemplars of changes in design. RI: Rhode Island.

In I1, the interview participants preferred messages to be written as if a real person was sending them. They also generally preferred a message tone that sounded like an adult or older sibling. The messages were edited in response to this feedback. I1 participants also requested additional reminders about the content of the in-person intervention. The team revised the links and created infographics (Figure 1). Most participants clicked on at least 1 link sent as part of a daily message. Interviewees across all three iterations said they only clicked on links that were personally relevant, visually appealing, and *“*something that’s quick.” Participants said they were more likely to click on links when they were having a bad day or were unfamiliar with the topic. In response, we revised the link descriptions to make topics seem more intriguing.

About one-third of all participants said they would prefer to receive the entire intervention through an app rather than an in-person component or text messages. The need for an in-person intervention was identified as a barrier to participation. Many said that their friends used *texting* exclusively through apps because of the lack of reliable cellular services.

### Feasibility and Acceptability: Quantitative Data Across Iterations

Of the 1064 daily queries sent to participants across all three iterations, 940 (88.34%) received a response. Participants responded to both questions (ie, how their day was going and whether cyberdrama had occurred in the past day) on 73.59% (783/1064) days. The rate of response to both daily questions improved from I1, during which, on average, participants replied to both queries on 61% (34/56) of days, to I2 (average of 46/56, 83% days) and I3 (average of 45/56, 81% days). Although we did see a small drop in engagement from I2 to I3, this discrepancy is not statistically significant; both engagement rates were significantly better compared with I1. All participants (n=17) who completed the 8-week follow-up survey rated the overall quality of iPACT as *good* or *excellent*. The ratings improved from I1 (7/7, 100% *good*) to I2 (2/4, 50% *good*; 2/4, 50% *excellent*) to I3 (1/7, 14% *good*; 6/7, 86% *excellent*). All participants who completed the 8-week survey agreed that they got the *kind of service* they wanted from iPACT, and all said they would come back to iPACT again if they could. Of the participants, 9 reported that they had already recommended the program to a friend or said they would do so.

## Discussion

### Principal Findings

To our knowledge, iPACT is the first SMS text message–based cyberbullying intervention for adolescents aged 13-17 years, specifically designed for initiation in clinical settings. Our application of an iterative design process through ongoing testing and refinement improved the qualitative and quantitative measures of success. By I3, participant engagement and ratings of acceptability and satisfaction increased: participants found the intervention *motivational* and highly rated both portions of the intervention [31].

Our findings support the importance of ongoing patient involvement in iterative intervention design [29]. Traditional intervention design requires full completion of the 8-week intervention and painstaking interview transcription and analysis before revising content and programming [28]. Our approach decreased the time required to make significant changes to both components of the program. Substantial changes included the addition of infographics, changes in daily SMS text message assessment programming, the addition of random text messages, and restructuring of the order of the SMS text message curriculum. The final iPACT intervention was designed to progressively and acceptably enhance participants’ ability to identify emotions, challenge and change negative thoughts and behaviors, engage in prosocial web-based habits, and support peers.

The area in which we were unable to adequately revise the intervention because of both scope and budget was participants’ desire for more personalized advice. Participants wanted to be able to solicit guidance from a live person when they became embroiled in a web-based conflict or were having a particularly bad day. This desire for a *just-in-time* adaptive intervention is not unique to our work [32]. Barriers to this real-time response include concerns about the need for 24/7 monitoring of messaging in case of human subjects’ concerns, lack of accurate automated identification of moments of conflict, and lack of budget to create this level of tailoring [33]. Research on how best to balance automated and human components in SMS text message–based mental health interventions is warranted [17,32].

Many participants requested delivery of the intervention through a smartphone app. Participants said their friends used apps for communication (ie, Facebook messenger, WhatsApp, and Snapchat) more often than SMS text messaging. Delivering the intervention through an app could integrate additional features such as games and quizzes, provide more nuanced on-demand content, and allow greater intervention personalization (eg, through background images). Apps may be feasible and acceptable in behavioral interventions. In future work, the iterative intervention refinement methods described herein could be relevant for developing app-delivered prevention content. The operating system–specific nature of mobile apps, however, as well as the need for internet data to function, may be important limitations.

Although the intervention topic was unrelated to patients’ pediatric visits, the intervention was initiated in a familiar setting where adolescents and parents often expect behavioral counseling to occur [9]. This location may enhance engagement. Future work should also consider the impact on clinical workflow and physicians’ and nurses’ perceptions of the program, as clinical staff engagement and buy-ins are essential for successful clinical implementation.

### Limitations

The findings should be appraised within the context of study limitations. The study was conducted at a single site using a convenience sample of participants. Certain groups of adolescents, including those in police and state agencies (eg, foster care or group home), were not eligible to participate because of institutional review board restrictions. Although this study demonstrates feasibility and acceptability, it may not be generalizable to other populations, given the small sample size of participants drawn from 1 city. The current design cannot speak to the program’s efficacy in reducing cybervictimization and its consequences; this would need to be measured in a randomized controlled trial.

### Comparison With Previous Work

Contrary to recent reports that participant engagement decreases over time, our data suggest that adolescents continued to engage with over the full 8-week period of I3. With an 88.34% (940/1064) response rate to any daily assessment and 84% (16/19) of participants completing postintervention interviews, iPACT had higher engagement and retention rates than many digital interventions [34-36]. By paying close attention to the participants’ input, the study team was able to add information that resonated with users [30,37]. During the follow-up interview, some participants reported using intervention elements to actively avoid web-based drama. However, it is important to note that the I3 arm had a very small sample size, and rates of engagement in a larger sample may not be the same, especially given that this sample was recruited in the context of a research study.

Although cyberbullying is equally common among youth of minority race or ethnicity and of low SES, few interventions have been developed specifically for this population [38]. These groups are less frequently included in research studies, and if and when they are included, they are often insufficient for *meaningful analyses* [39]. Cybervictimization interventions developed with largely White or high-SES populations may need tailoring for minority or low-SES populations because of differences in social norms, prevalence of in-person violence and disenfranchised neighborhoods, and underlying racism and mistrust [40]. This study recruited a sample of predominantly minority and low-SES adolescents. Engaging in the collaborative design and refinement process with this group is paramount.

### Conclusions

Agile qualitative methods were used to iteratively develop iPACT, a mixed-modality digital intervention for adolescents affected by cybervictimization. Improvements in participants’ satisfaction and level of daily participation suggest the importance of agile development methods and suggest the acceptability of initiating digitally augmented preventive interventions in the pediatric primary care setting. Future work should investigate the efficacy of iPACT in preventing cybervictimization and its consequences.

## Abbreviations

I1: iteration 1
I2: iteration 2
I3: iteration 3
iPACT: Intervention to Prevent Adolescent Cybervictimization with Text message
SES: socioeconomic status
